# Progression of erectile function in men with chronic obstructive pulmonary disease: a cohort study

**DOI:** 10.1186/s12890-019-0902-y

**Published:** 2019-09-02

**Authors:** Eui Geum Oh, Jae Yong Yoo

**Affiliations:** 1 Mo-Im Kim Nursing Research Institute, Yonsei Evidence Based Nursing Centre of Korea, a Joanna Briggs Institute Centre of Excellence, College of Nursing, Yonsei University, 50-1 Yonsei-ro, Seodaemun-gu, Seoul, 03722 Korea; 2 Department of Nursing, College of Medicine, Chosun University, Gwagnju, Korea, 309 Pilmundae-ro, Dong-gu, Gwangju, 61452 Korea

**Keywords:** Chronic obstructive pulmonary disease, Longitudinal study, Male, Erectile dysfunction

## Abstract

**Background:**

Although sexual function is a quality of life aspect that is markedly affected in males with chronic obstructive pulmonary disease (COPD), this topic has not attracted much attention and research on this matter is lacking. In this study, we investigated longitudinal changes in the erectile function of men with COPD in order to identify latent groups and influencing factors.

**Methods:**

A total of 185 men with COPD from the Korean Obstructive Lung Disease study, which was conducted from 2005 to 2013, were analyzed in this study. Data on their erectile function, based on the International Index of Erectile Function-5, were collected over a period of 4 years. Growth mixture modeling and logistic regression analysis were used to determine the factors predicting distinct erectile function changes over time.

**Results:**

Overall, subjects’ erectile function slightly improved in the first year and then gradually worsened over time. Using growth mixture modeling, we identified four distinct latent groups, which we labeled as follows: “consistently maintained normal erectile function” (9.7%), “rapidly worsened and then rapidly improved” (9.2%), “gradually improved in the early stage and then gradually worsened” (36.8%), and “consistently maintained poor erectile function” (44.3%). Progression of erectile function was significantly associated with age, economic status, and self-rated health status.

**Conclusions:**

This suggests that comprehensive patient care involving the management of COPD as well as erectile dysfunction in patients with chronic respiratory disease is important from a prophylactic perspective and should be developed in accordance with the characteristics of the disease process.

## Background

Chronic obstructive pulmonary disease (COPD) is a progressive disorder characterized by airflow limitation that is not fully reversible, and is accompanied by chronic inflammation of the airway and lungs [1]. In 2016, COPD was the third most common cause of death worldwide [2]. In Asia in particular, the rate of increase in the incidence of COPD is exacerbated by high smoking rates; exposure to indoor and outdoor air pollution arising from biomass fuel combustion; and frequent exposure to yellow dust, particulate matter, and industrial dust [3]. The global COPD prevalence rates are estimated to be 7.5–10.3%, whereas in Korea, the prevalence is reported to be 13.7% (men: 23.3%, women: 6.5%) among individuals aged > 40 years, according to the 5th Korea National Health and Nutrition Examination Survey [4]. The considerably higher COPD prevalence rate in Korea is thought to be related to high smoking rates and exposure to tuberculosis, which are both risk factors for COPD, along with a rapidly aging population [3–5]. Although the smoking rate among Korean adults decreased from 66.7% in 1995 to 36.2% in 2013, it remains the highest among Organization for Economic Co-operation and Development (OECD) countries [4], and Korea has the highest rate of tuberculosis outbreaks and deaths among OECD countries [3].

The relatively high rate of COPD in Korea poses a financial burden due to COPD-related health and medical expenses, and leads to deterioration in the quality of life (QoL). The ultimate goal of health management for patients with COPD is to improve their QoL, a major subjective marker of health status that is used to assess the effects of treatment and interventions [3, 6]. Men exhibit an increased prevalence of COPD after 60 years of age [7, 8], and sexual function is a QoL aspect that is markedly affected in men with COPD [9, 10]. The mechanism underlying sexual dysfunction in COPD patients is not yet fully understood. Smoking, chronic hypoxia, aging, systemic/endothelial inflammation, hormonal imbalances (hypogonadism or lower testosterone levels), decreased physical activity, fear of breathing difficulty, cardiovascular disease, and medication side effects are considered to be possible contributing factors [11]. Respiratory or physical symptoms, such as dyspnea, cough, weakness, and reduced physical activity affect both erectile function and sexual activity, which lowers the QoL of COPD patients. For many patients, the loss of erectile function triggers a sense of shame, as they perceive that they can no longer function as men, which can reduce their self-esteem and cause depression [12].

Despite these detrimental consequences, the effect of COPD on erectile function in men is underappreciated in clinical practice, partly because patients often feel uncomfortable talking about their erectile function with healthcare providers [7, 9, 10]. In Korea and other Asian countries, discussion of sexual issues is a cultural taboo: only 2% of men and women in Korea discuss their sexual health problems with medical personnel [13]. Therefore, information regarding erectile dysfunction among male patients with COPD is sparse and erectile dysfunction tends to be underreported in Korea relative to other countries [3, 14, 15]. According to the Massachusetts Male Aging Study, a large epidemiological study performed in the US, 52% of 1,709 men aged 40–70 years experienced sexual dysfunction at some point during their life [16]. In an epidemiological study of > 10,000 men aged 20–75 years in five Asian countries, including Korea (the Asian Men’s Attitudes to Life Events and Sexuality study), researchers found that the rate of self-reported sexual dysfunction was 6.4% [15].

Sexual function is an indicator of health and QoL, and it changes over time. However, previous studies have primarily investigated the prevalence of erectile or sexual function and the correlations with patient characteristics [9, 10, 17]. Furthermore, most of these studies were cross-sectional; therefore, researchers cannot fully explain how patient characteristics affect progression in erectile function. Since the physical functioning of patients with COPD gradually deteriorates over time, it is important to understand these dynamic changes in health status and to plan patient-centered care and management accordingly [18].

Therefore, in this study we used the Korean Obstructive Lung Disease (KOLD) cohort data to investigate the patterns of change over time in the erectile function of men with COPD. We aimed to identify factors that affect these patterns, as a basis for the development of strategies for patient-centered care and intervention programs. The following research questions were addressed:

1. Are there latent categories among male patients with COPD, defined by distinct longitudinal patterns of erectile function levels?
2. How many latent groups exist, and what are the associations between distinct erectile function patterns and subjects’ socio-demographic, disease-related physical/functional status, and cognitive variables?
3. If there are multiple baseline erectile functions, what are the putative factors for discriminating these functions?
4. If there are multiple distinct patterns, what are the factors that affect longitudinal patterns?

## Methods

### Study design

This study was a secondary analysis of the data of 185 men with COPD from the KOLD cohort. The KOLD cohort was established by 16 secondary or tertiary hospitals in South Korea between 2005 and 2013, for the purpose of building a systematic diagnostic model of COPD and detecting comprehensive predictors of COPD.

### Subjects

The following subjects were included in the study: men aged ≥40 years, with chronic respiratory symptoms, and who were diagnosed with COPD by a pulmonary physician according to the Global Initiative for Chronic Obstructive Lung Disease (GOLD) guidelines [1]. Subjects were excluded if they 1) had been diagnosed with a second serious health conditions (e.g., heart failure, severe cerebrovascular disease, chronic urological disease, or renal failure); 2) were taking medications or participating in a separate program that affected erectile function; and 3) had undergone lung surgery, such as a lobectomy. A total of 144 patients were excluded because they did not undergo pulmonary function testing at baseline, or had fewer than two follow-up erectile function values. Thus, 185 of the 329 original patients comprised the subjects of this study.

### Measurements

The erectile function of men with COPD was assessed using the Korean version of the International Index of Erectile Function-5 (IIEF-5) [19]. The IIEF-5 is a self-administered questionnaire scored on a five-point Likert scale that contains five items related to erectile and orgasmic functioning, and its validity and reliability are internationally recognized [20]. Examples of the items in the IIEF-5 include: “During sexual intercourse, how difficult was it to maintain your erection to completion of intercourse?” and “When you attempted sexual intercourse, how often was it satisfactory for you?” The total score ranges from 1 to 25 points. The subjects were classified into five sexual function groups according to Rosen’s cutoff value [20]: none (IIEF-5 score: 25–22), mild (21–17), mild-moderate (16–12), moderate (11–8), and severe (7–1). In this study, Cronbach’s alpha for the IIEF-5 tool was 0.86. In cases wherein self-reporting was difficult or rejected, a trained researcher collected the necessary data via interview.

This study was based on the “Chronic illness trajectory model” by Corbin and Strauss [21]. In this model, chronic diseases are not fixed, but are considered to be a process that can unfold with various trajectories or patterns over time. Socio-demographic, disease-related physical/functional, and cognitive factors may be associated with longitudinal changes in individuals’ health levels. In this model, it is important for the patient, family, and health professionals to understand and manage the perceptions of the individual and the contextual situations, treatment/care plans, and disease progression that affect the chronic disease status. Based on this model, the following independent variables were measured using structured questionnaires and clinical test results. Demographic characteristics included age, education level, smoking status, current medications, and economic status. Symptom variables included the number of respiratory symptoms, sleep disturbance, and the level of anxiety and depression, as measured by the Beck Anxiety Inventory (BAI) [22]. The BAI consists of 21 multiple-choice questions regarding anxiety symptoms that the subject may have experienced during the past month (e.g., “How much you have been bothered by fear of losing control?”). The Beck Depression Inventory (BDI) consists of 21 items designed to evaluate symptoms and attitudes associated with depression, such as “I feel the future is hopeless and that things cannot improve.” The Beck items are scored on a four-point scale ranging from 0 to 3, with a higher score indicating higher severity. Self-rated health was measured by a single item; “What do you think of your overall health at present?” Responses were scored as 0 (excellent), 1 (good), 2 (fair), 3 (poor), and 4 (very poor), with a higher score indicating a poorer status.

Functional variables included pulmonary function; the Body mass index, airflow Obstruction, Dyspnea, and Exercise capacity (BODE) index, a multidimensional prognostic tool for COPD [23]; disease severity; number of comorbidities; and exacerbations in the last year. Pulmonary function tests were performed using spirometry (2130 or Vmax 22, SensorMedics, Yorba Linda, CA; PFDX, MedGraphics, Saint Paul, MN, USA), according to the American Thoracic Society (ATS) guidelines [1], and evaluated the forced vital capacity (FVC), forced expiratory volume in 1 s (FEV_1_), and ratio of FEV_1_ to FVC (FEV_1_/FVC). The BODE index ranges from 0 to 10, with a higher score indicating a poorer status. Airflow obstruction status was evaluated using the FEV_1_ value. Dyspnea was assessed using the Modified Medical Research Council Dyspnea Scale, and exercise capacity was measured by the six-minute walk distance test, according to the ATS guidelines [24]. The subjects were scored from 0 to 3 for each threshold value. COPD severity was assessed using the GOLD guidelines [1, 6].

### Data collection and statistical analysis

All data were obtained at baseline and subsequently every 12 months over a 4-year period by trained clinical researchers using the ATS standardized guidelines [1]. Researchers at each of the 16 participating institutions were trained to collect data according to the standardized data collection protocol. Research supervisors periodically supervised quality control of the data collection.

We hypothesized that individuals could be clustered into smaller groups according to the patterns of change in the erectile function status [25]. We applied growth mixture modeling (GMM) to identify patterns of change using the longitudinal cohort data [26]. GMM is based on a conventional growth model involving the estimation of growth representative of the entire population. However, the patterns of change in the erectile function levels of COPD patients may not be the same for all patients. Nylund et al. have emphasized that certain subgroups of individuals exhibit growth trajectories (reflected in the model parameters) that differ significantly from the estimated trajectory of the population [27]. We used STATA 12.0 to construct a GMM with linear and quadratic effects of time to distinguish latent subgroups according to patterns of change in the erectile function levels of the COPD patients. An analysis was then performed to determine the factors influencing subgroup membership.

The statistical analyses were performed in three stages, as follows: In the first stage, using the IIEF-5 scale as a continuous measure, GMM was used to identify naturally occurring trajectories in the changes in erectile function over time. In the GMM, we used all available IIEF-5 scores for each of the participants at five time points (baseline, and 12, 24, 36, and 48 months later) to identify subgroups of individuals with relatively similar trajectories. For each distinct latent group, different intercepts (i.e., baseline values) and slope parameters (reflecting patterns of change over time) were estimated to describe the longitudinal patterns in outcome measures. One major benefit of GMM is that it can model non-linear and linear trajectories [25]. We used cubic polynomials to model the shape of each latent trajectory as follows:

$$ Erectile\ function=I+{a}_1{T}^1+{a}_2{T}^2+{a}_3{T}^3, $$

where *Erectile function* is the predicted IIEF-5 score, *I* is the predicted intercept, *T* is the time at which the variable is measured, and *a*_*1*_*, a*_*2*_*,* and *a*_*3*_ are linear, quadratic, and cubic parameters, respectively.

We used unconditional models, in which no independent variables were included, to determine the optional number of latent erectile function groups for describing the patterns of change. We increased the number of groups sequentially to a total of five and compared the resulting models. There was no absolute criterion for determining how many groups to include in the GMM; however, the optimal number of latent groups can be determined by considering model fit criteria. Therefore, we considered the Akaike Information Criterion (AIC) and the Bayesian Information Criterion (BIC) [25, 26]. Following Nagin’s approach [26], the smaller the absolute values of the AIC and BIC, the better the model. Entropy is another good indicator, because the probability of belonging to each latent group approaches 1. In addition to comparing models based on these criteria, when finally classifying potential groups, we required the minimum sample size in each group to be 10, which is clinically meaningful, as a model with too few cases in a subgroup is not reliable [25].

In the second statistical analysis stage, we tested for differences between groups with distinct erectile function patterns with regard to patients’ socio-demographic, disease-related physical/functional status, and cognitive variables. For descriptive statistics, we used the mean and standard deviation for continuous variables and proportions for categorical variables. To compare the characteristics of the subjects across latent groups, categorical variables were analyzed using chi-square tests, and continuous variables were analyzed using analysis of variance and post-hoc Scheffe’s test.

In the third statistical analysis stage, two logistic regression analyses were conducted to identify correlates of baseline erectile function and factors predicting different erectile function trajectories during the observation period. Socio-demographic, physical/functional, and cognitive factors were included as independent variables.

In the first logistic regression analysis, ordinal logistic regression was used to determine the influence of putative predictive factors on baseline erectile function. In practice, the baseline erectile function level of a subject is determined at the time of enrollment in a medical facility or at the beginning of COPD treatment. Based on these baseline values, patients with COPD with erectile function issues can be identified early and access to individualized interventions can be ensured. In the second logistic regression analysis, multinomial logistic regression was performed to assess the influence of factors predicting the progression in the erectile function. These factors can guide treatment strategies for improving long-term health status and sexual function-related symptoms, leading to improved symptoms and QoL. The results are summarized as odds ratios (ORs) with 95% confidence intervals (CIs). *P* < 0.05 was considered statistically significant. SPSS 22.0 and STATA 12.0 were used for data analysis. The entire analysis of this study was conducted under the consultation and review of biostatisticians.

### Ethics

This cohort protocol was approved by the institutional review board of all participating hospitals (Approval No 2005–0345). All subjects were provided with oral and written explanations of the study processes. Written informed consent was provided by each participant.

## Results

### General characteristics of subjects

The subjects’ characteristics are summarized in Table 1. The mean age of the subjects was 65.4 ± 7.3 years (range: 45–84 years), and 108 (58.4%) subjects were aged ≥65 years. Fifty-nine (31.9%) subjects were current smokers, and the mean number of smoking pack-years was 22.6 ± 12.8. Over 80% of the patients had moderate to severe ventilator defects, based on the FEV_1_ value. According to the combined COPD assessment criteria (symptoms, risk of exacerbation, and lung function test results) [6], 37.8% of patients were classified as GOLD A, 24.3% as GOLD B, 10.8% as GOLD C, and 27.0% as GOLD D.

**Table 1:** General characteristics of the subjects at baseline (*n* = 185)

Characteristics (continuous variables)	Mean	SD
Age (years)	65.4	7.3
BMI (kg/m^2^)	23.1	3.3
Income (US dollars/month)	2,230.9	1,850.2
Smoking history (pack year)	22.6	12.8
Number of comorbidities	0.5	0.4
Number of respiratory symptoms	2.6	1.4
Number of exacerbation in last year	0.77	2.45
FEV_1_ (L)	1.62	0.53
FEV_1_ (predicted %)	59.94	18.34
FVC (L)	3.45	0.78
FEV_1_/FVC (%)	46.84	10.66
6MWD (m)	447.2	78.4
mMRC dyspnea scale score	1.5	0.9
BODE index score	2.08	1.79
BAI score	6.01	7.55
BDI score	9.10	9.37
Self-rate health status	2.25	0.78
IIEF-5 score at baseline	10.79	5.30
IIEF-5 score at 12 months	10.92	5.28
IIEF-5 score at 24 months	9.68	5.69
IIEF-5 score at 36 months	8.45	4.38
IIEF-5 score at 48 months	7.72	4.39
Characteristics (categorical variables)	n	%
Education level		
≥ bachelor’s degree	41	22.1
high school	66	35.7
≤ middle school	78	42.2
Current smoking		
yes	59	31.9
no	126	68.1
Current medication		
yes	134	72.4
no	51	27.6
Sleep disturbance		
yes	53	28.6
no	132	71.4
Emergency room visit in last year		
yes	35	18.9
no	150	81.1
Severity of COPD based on		
combined COPD assessment		
GOLD A (low risk, fewer symptoms)	70	37.8
GOLD B (low risk, more symptoms)	45	24.3
GOLD C (high risk, fewer symptoms)	20	10.8
GOLD D (high risk, more symptoms)	50	27.0
Baseline erectile function (IIEF-5 score)		
Normal (25–22)	14	7.6
Mild (21–17)	33	17.7
Mild-moderate (16–12)	41	22.2
Moderate (11–8)	26	14.1
Severe (≤7)	71	38.4

*SD* standard deviation, *BMI* body mass index, *FEV*_*1*_ forced expiratory volume in 1 s, *FVC* forced vital capacity, *6MWD* six-minute walk test, *mMRC* modified Medical Research Council, *BODE* body mass index (B), the degree of airflow obstruction (O), functional dyspnea (D), exercise capacity (E) index, *GOLD* global initiative for chronic obstructive lung disease, *IIEF-5* International index of erectile function-5, *COPD* chronic obstructive pulmonary disease, *BAI* beck anxiety inventory, *BDI* beck depression inventory

We administered a questionnaire that included a question on whether pharmacological therapy for COPD treatment was provided. The researchers at the cohort-registered hospital reviewed the patients’ medical records to determine the type of medication currently being administered. At the time of cohort registration, 72.4% (*n* = 134) of the patient were receiving pharmacological treatment, namely beta-2 agonists, muscarinic antagonists, inhaled corticosteroid/long acting beta-2 agonists, methylzanthines, and mucolytics. We assumed the influence of any changes in these medications during the study period to be minimal.

The mean post-bronchodilator FEV_1_ was 59.9 ± 18.3%, and the mean FEV_1_/FVC was 46.8 ± 10.6% of the predicted values. The mean baseline BODE index and IIEF-5 scores were 2.08 ± 1.79 and 10.7 ± 5.3, respectively. According to the IIEF-5 criteria [20], 7.6% individuals had normal, 17.7% had mild, 22.2% had mild–moderate, 14.1% had moderate, and 38.4% had severe erectile dysfunction.

### Progression of erectile function

Notably, 92.4% of the patients had mild to severe erectile dysfunction at baseline. Although the erectile function of these patients partially improved during the first year after their registration in the cohort, it tended to worsen thereafter. The estimated intercept of erectile function was 11.6 (standard error [SE] = 0.6), and the slope was − 0.7 (SE = 0.1) for all subjects. Despite some fluctuation, these values indicated that erectile function gradually worsened over time. In the GMM analyses with different numbers of latent groups, the AIC and BIC values were the lowest for four latent groups. The possible models of erectile function trajectories, with the number of subjects in each group and model fit indices, are summarized in Table 2.

**Table 2:** Model fit statistics and parameter estimates for distinct trajectories of erectile function (IIEF-5)

Group	n	AIC	BIC	Log- likelihood	Intercept (SE)	Linear slope (SE)	Quadratic slope (SE)
1	185	2640.77	2648.82	− 2635.77	11.685^***^ (0.605)	− 0.725^***^ (0.193)	0.011 (0.161)
2	107	2512.49	2528.59	− 2502.49	7.515^***^ (0.671)	− 0.737^**^ (0.197)	0.143 (0.169)
	78				17.506^***^ (0.770)	−0.690^*^ (0.235)	−0.278 (0.203)
3	86	2479.59	2503.74	− 2464.59	6.018^***^ (0.697)	− 0.594^**^ (0.206)	0.126 (0.173)
	78				15.346^***^ (0.698)	−1.026^***^ (0.215)	−0.198 (0.183)
	21				21.297^***^ (1.414)	−0.188 (0.411)	− 0.170 (0.325)
4	82	2461.49	2501.75	− 2470.84	5.152^***^ (0.823)	− 0.395 (0.231)	−0.112 (0.180)
	17				16.300^***^ (2.090)	−2.260^***^ (0.716)	2.438^***^ (0.523)
	18				21.679^***^ (1.330)	−0.193 (0.410)	− 0.155 (0.311)
	68				14.706^***^ (1.188)	−0.416 (0.392)	− 0.632^**^ (0.223)
5	22	2470.84	2503.04	− 2461.49	5.153^**^ (2.345)	−0.395 (0.630)	−1.527^***^ (0.359)
	56				5.152^***^ (1.181)	− 0.395 (0.323)	− 0.079 (0.177)
	36				16.300^***^ (2.101)	−2.260^**^ (0.718)	0.076 (0.305)
	52				14.706^***^ (1.190)	−0.416 (0.393)	2.377^***^ (0.458)
	19				21.679^***^ (1.339)	−0.194 (0.411)	−0.146 (0.311)
Selected model Group name						n (%)	
Four group models		Group A: consistently good erectile function 18 (9.7) Group B: rapidly worsened and then rapidly improved 17 (9.2) Group C: gradually improved and then gradually worsened 68 (36.8) Group D: consistently poor erectile function 82 (44.3)					

*IIEF-5* International index of erectile function-5, *AIC* akaike information criterion, *BIC* Bayesian information criterion, *SE* standard error

*p < .05, **p < .01, ***p < .001

**Fig. 1:**
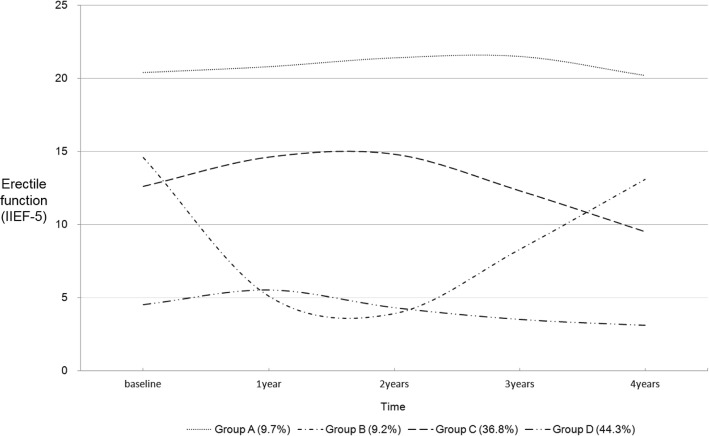
Four latent groups of longitudinal erectile function patterns

The four groups were defined according to the observed patterns of change: “Group A” consistently maintained normal erectile function (9.7%, *n* = 18), “Group B” had erectile function that rapidly worsened and then rapidly improved (9.2%, *n* = 17), “Group C” had erectile function that gradually improved during the early stage, but then gradually worsened over time (36.8%, *n* = 68), and “Group D” consistently had poor erectile function (44.3%, *n* = 82) (Fig. 1). Differences in the subjects’ characteristics according to the four latent groups are summarized in Table 3.

**Table 3:** Differences in patient characteristics according to four latent groups

Variables	Category	Group A	Group B	Group C	Group D	F / χ^2^ (*p*)
		Consistently good (*n* = 18, 9.7%)	Rapidly worsened & rapidly improved (n = 17, 9.2%)	Gradually improved & gradually worsened (n = 68, 36.8%)	Consistently poor (n = 82, 44.3%)	
Age (mean ± SD, years)		59.9 ± 6.0	66.3 ± 7.9	62.0 ± 6.6	69.2 ± 5.9	20.447 (< 0.001) ^D > C > A^
BMI (kg/m^2^)		24.1 ± 2.6	23.2 ± 4.1	23.9 ± 3.0	22.3 ± 3.4	3.724 (0.012) ^A > C^
Obesity (n, %)	normal	12 (66.4)	10 (58.8)	42 (61.8)	60 (73.2)	15.751 (0.072)
	under weight	0 (0.0)	2 (11.8)	2 (2.9)	10 (12.2)	
	over weight	6 (33.3)	4 (23.5)	22 (32.4)	10 (12.2)	
	obese	0 (0.0)	1 (5.9)	2 (2.9)	2 (2.4)	
Education (n, %)	≥bachelor	7 (38.9)	3 (17.6)	9 (13.2)	22 (26.8)	28.301 (0.020)
	high school	8 (44.4)	7 (41.7)	30 (44.1)	21 (25.6)	
	≤middle school	3 (16.7)	7 (41.7)	29 (42.7)	39 (47.6)	
Income (n, %)	≥2,000	14 (77.8)	8 (47.1)	31 (45.6)	32 (39.0)	9.844 (0.020)
(US dollars /month)	< 2,000	4 (22.2)	9 (52.9)	37 (54.4)	50 (61.0)	
Current smoker	yes	6 (33.3)	4 (23.5)	25 (36.8)	24 (29.3)	1.568 (0.667)
	no	12 (66.7)	13 (76.5)	43 (63.2)	58 (70.7)	
Number of comorbidities		0.67 ± 0.68	0.66 ± 0.61	0.62 ± 0.52	0.62 ± 0.68	1.991 (0.117)
FEV_1_ (L)		2.01 ± 0.59	1.58 ± 0.44	1.75 ± 0.52	1.44 ± 0.47	8.527 (< 0.001) ^A > C,D^
FEV_1_ predicted (%)		68.49 ± 18.10	59.23 ± 17.83	61.83 ± 19.11	56.64 ± 17.34	2.492 (0.062)
FVC (L)		3.76 ± 0.78	3.24 ± 0.56	3.67 ± 0.85	3.25 ± 0.69	5.368 (0.001) ^A > C^
FEV_1_/FEV ratio (%)		53.19 ± 10.43	49.33 ± 13.02	47.95 ± 10.71	44.01 ± 9.36	4.898 (0.003) ^A > D^
6MWD (m)		476.1 ± 51.8	439.5 ± 47.2	473.0 ± 77.6	420.7 ± 80.9	7.045 (< 0.001) ^A > B,D^
Number of symptoms		2.72 ± 1.27	2.88 ± 1.17	2.56 ± 1.46	2.65 ± 1.36	0.276 (0.843)
Dyspnea (mMRC) score		1.22 ± 0.94	1.65 ± 1.22	1.47 ± 0.94	1.67 ± 0.99	1.260 (0.290)
BODE index score		1.17 ± 1.30	2.29 ± 1.93	1.78 ± 1.57	2.48 ± 1.93	3.804 (0.011) ^D > A^
Sleep disturbance (n, %)	yes	4 (22.2)	6 (35.3)	23 (33.8)	20 (24.4)	2.349 (0.503)
	no	14 (77.8)	11 (64.7)	45 (66.2)	62 (75.6)	
Exacerbation (n, %)	yes	2 (11.1)	4 (23.5)	10 (14.7)	19 (23.2)	2.704 (0.440)
in last year	no	16 (88.9)	13 (76.5)	58 (85.3)	63 (76.8)	
BAI score		5.83 ± 5.80	6.27 ± 5.74	5.79 ± 8.67	6.17 ± 7.32	0.042 (0.989)
BDI score		8.00 ± 9.71	7.76 ± 8.99	8.07 ± 7.74	10.48 ± 10.53	1.059 (0.368)
Self-rated health status		1.89 ± 0.76	2.18 ± 0.88	2.25 ± 0.70	2.35 ± 0.81	1.858 (0.138)

*SD* standard deviation, *FEV*_*1*_, forced expiratory volume in 1 s, *FVC* forced vital capacity, *6MWD* six-minute walk test, *mMRC* modified Medical Research Council, *BODE* body mass index (B), the degree of airflow obstruction (O), functional dyspnea (D), exercise capacity (E) index, *BAI* beck anxiety inventory, *BDI* beck depression inventory, *BMI* body mass index

### Factors affecting baseline erectile function

The four groups were further divided into three clusters according to the baseline erectile function level: Cluster 1 (normal baseline function, Group A, 9.7%, n = 18), Cluster 2 (mild to moderate dysfunction at baseline, Groups B and C, 46.0%, *n* = 85), and Cluster 3 (severe dysfunction at baseline, Group D, 44.3%, n = 82). Cluster 1 was set as the reference cluster for the ordinal logistic regression. Factors that increased the likelihood of belonging to a higher-dysfunction cluster at baseline were older age (OR 1.14, 95%CI 1.08–1.21), lower economic status (OR 1.27, 95%CI 1.05–1.57), higher BODE index (OR 1.31, 95%CI 1.02–1.68), and poorer self-rated health status (OR 1.69, 95%CI 1.01–2.85) (Table 4).

**Table 4:** Results of ordinal logistic regression analysis of factors affecting the baseline erectile function level

Variables	Category	Cluster 1 (normal baseline, Group A) ^Reference^	Cluster 2 (mild to moderate baseline, Group B & C)	Cluster 3 (severe baseline, Group D)	Cluster 1 vs. Cluster 2 and 3		
		(n = 18, 9.7%)	(n = 85, 46.0%)	(n = 82, 44.3%)	Odd ratio	95% CI	*p*
Age (mean ± SD, years)		59.9 ± 6.0	62.9 ± 7.1	69.2 ± 5.9	1.14	1.08–1.21	< 0.001
Education (n, %)	≥bachelor’s degree	7 (38.9)	12 (14.1)	22 (26.8)	0.96	0.58–1.62	0.821
	high school	8 (44.4)	37 (43.5)	21 (25.6)			
	≤middle school	3 (16.7)	36 (42.4)	39 (47.6)			
Income (n, %)	≥2,000	14 (77.8)	39 (45.9)	32 (39.0)	1.27	1.05–1.57	0.021
(US dollars/month, n)	< 2,000	4 (22.2)	46 (54.1)	50 (61.0)			
Current smoker (n, %)	yes	6 (33.3)	29 (34.1)	24 (29.3)	0.95	0.47–1.92	0.905
	no	12 (66.7)	56 (65.9)	58 (70.7)			
Number of comorbidities		0.67 ± 0.68	0.65 ± 0.61	0.62 ± 0.68	1.17	0.72–1.78	0.527
Number of symptoms		2.72 ± 1.27	2.62 ± 1.40	2.65 ± 1.36	0.93	0.74–1.12	0.596
BODE index sore		1.17 ± 1.30	1.88 ± 1.65	2.48 ± 1.93	1.31	1.02–1.68	0.032
Sleep disturbance (n, %)	yes	4 (22.2)	29 (34.1)	20 (24.4)	0.64	0.29–1.39	0.265
	no	14 (77.8)	56 (65.9)	62 (75.6)			
Exacerbation (n, %)	yes	2 (11.1)	14 (16.5)	19 (23.2)	0.88	0.35–2.19	0.787
in last year	no	16 (88.9)	71 (83.5)	63 (76.8)			
BAI score		5.83 ± 5.80	5.89 ± 8.14	6.17 ± 7.31	1.02	0.96–1.08	0.519
BDI score		8.00 ± 9.71	8.01 ± 7.95	10.48 ± 10.53	0.99	0.94–1.04	0.753
Self-rated health status		1.89 ± 0.76	2.24 ± 0.73	2.35 ± 0.81	1.69	1.01–2.85	0.047

*SD* standard deviation, *BODE* body mass index (B), the degree of airflow obstruction (O), functional dyspnea (D), exercise capacity (E) index, *BAI* beck anxiety inventory, *BDI* beck depression inventory, *CI* confidence interval

### Factors affecting progression of erectile function

Multinomial logistic regression was performed to investigate predictors of the progression of erectile dysfunction. Group A was again set as the reference group. Distinct patterns were significantly associated with age (Group B vs. A: OR 1.23, 95%CI 1.04–1.44; Group D vs. A: OR 1.26, 95%CI 1.10–1.45), economic status (Group C vs. A: OR 1.22, 95%CI 1.11–1.32; Group D vs. A: OR 1.28, 95%CI 1.12–1.53), and self-rated health status (Group B vs. A: OR 5.59, 95%CI 1.13–2.76; Group C vs. A: OR 5.57, 95%CI 1.41–2.19; Group D vs. A: OR 6.67, 95%CI 1.62–2.75) (Table 5).

**Table 5:** Results of multinomial logistic regression analysis of factors affecting the longitudinal pattern of erectile function

Variables	Group A vs. Group B			Group A vs. Group C			Group A vs. Group D		
	Odd ratio	95% CI	*p*	Odd ratio	95% CI	*p*	Odd ratio	95% CI	*p*
Age	1.23	1.04–1.44	0.013	1.03	0.91–1.16	0.622	1.26	1.10–1.45	0.001
Education	0.92	0.66–1.21	0.545	0.90	0.72–1.43	0.588	0.88	0.57–1.33	0.432
Income	0.99	0.98–0.99	0.006	1.22	1.11–1.32	0.003	1.28	1.12–1.53	0.005
Current smoking	0.85	0.13–5.59	0.868	1.53	0.36–6.44	0.561	1.32	0.28–6.04	0.722
Number of comorbidities	1.22	0.92–1.67	0.452	1.02	0.96–1.23	0.744	1.34	0.98–1.72	0.343
Number of symptoms	0.96	0.49–1.93	0.917	0.70	0.39–1.26	0.241	0.78	0.43–1.42	0.424
BODE index score	1.72	0.83–3.58	0.141	1.16	0.62–2.18	0.634	1.71	0.90–3.25	0.901
Sleep disturbance	1.23	0.15–9.90	0.848	1.15	0.19–6.94	0.876	0.66	0.10–4.27	0.669
Exacerbation in last year	0.82	0.43–1.52	0.528	0.96	0.74–1.24	0.774	1.03	0.78–1.36	0.822
BAI score	1.07	0.92–1.23	0.390	1.01	0.90–1.14	0.789	1.03	0.91–1.17	0.593
BDI score	0.93	0.79–1.07	0.330	0.94	0.83–1.05	0.267	0.95	0.83–1.07	0.423
Self-rated health status	5.59	1.13–27.6	0.034	5.57	1.41–21.9	0.014	6.67	1.62–27.5	0.009

*BODE* body mass index (B), the degree of airflow obstruction (O), functional dyspnea (D), exercise capacity (E) index, *BAI* beck anxiety inventory, *BDI* beck depression inventory, *CI* confidence interval

## Discussion

This study investigated the progression of the erectile function of men with COPD over the course of 4 years, in order to identify latent groups and determine the influencing factors. The men with COPD included in this study exhibited slight improvement in the total mean erectile function score during the first year, but this score tended to worsen gradually over time. We identified four distinct latent groups based on individual changes in erectile function over time.

Sexual dysfunction in elderly patients with chronically weakened physical and mental conditions has not received much attention [7, 9, 13]. In addition, there are social prejudices and a lack of understanding of sexual behavior and satisfaction in patients with chronic illness who still experience sexual desire [10, 13, 17]. Men with COPD experience significantly lower erectile function and less sexual satisfaction than do men without COPD, making it difficult to have a healthy sexual life; in turn, erectile dysfunction hinders disease management and can result in depressive symptoms and reduced QoL [10, 28]. Collins’ study of 90 patients with moderate to severe COPD revealed that ≥74% of the patients had sexual health problems [7]. In a Taiwanese cohort study of 57,928 participants [28], patients with COPD had 1.88-fold more sexual problems than did patients without COPD. These findings suggest that active care and management of erectile function and sexual satisfaction is required in men with COPD.

Additionally, the prevalence and severity of COPD increases with age [4, 5, 8]. We found that subjects in the persistently poor erectile function group had a higher mean age than those in the persistently good erectile function group. Previous studies have also reported an association between age and sexual function in patients with chronic illness [7, 9, 12]. The recently revised guidelines for COPD management emphasize the importance of regular assessment and management of subjective health status [6]. This includes verifying the symptoms subjectively experienced by the patient and identifying the degree of their impact on daily life by means of a COPD assessment test (CAT) [6]. Therefore, outpatients or hospitalized COPD patients should be encouraged to utilize subjective patient assessment tools, such as the CAT and IIEF-5, and to undergo objective clinical tests, such as laboratory tests or pulmonary function tests. When a physician or a clinical nurse first assesses elderly male COPD patients, erectile function evaluation using the IIEF-5 should be considered essential. Healthcare professionals should not overlook the area of sexual health, as it is an important aspect of QoL in older patients. Erectile dysfunction is a problem that can negatively affect sexual satisfaction for both men and women. However, most previous studies have focused on male impotence, and only two studies included women with COPD [17, 29]. Kaptein et al.’s study [17] also revealed that more than two-thirds of patients do not express their thoughts regarding sexual function to their physicians or partners. Attention should be afforded to the sexual function and sexual satisfaction of COPD female patients as well as male patients, and these subjects should be investigated in future studies.

The erectile function of the subjects included in the current study tented to improve in the first year and then deteriorate again. As there have been no prior studies on the longitudinal analysis of erectile function in male COPD patients, various interpretations are possible. The subjects included in the study were first diagnosed with COPD by a physician and enrolled in the cohort, and then visited outpatient clinics of medical facilities regularly to receive optimal treatment including non-pharmacological treatments. In this study, the GOLD A group with few symptoms comprised 37.8% of the patients, and 68.2% corresponded to GOLD stage 1 and 2 based on the FEV_1_ values. Therefore, it is likely that interventions such as health education and correction of health-related lifestyle habits including smoking cessation were more frequent in the first year of diagnosis. This may also be the result of the limitations of self-administered questionnaires. If the subjects in the cohort report severe erectile dysfunction, this may be the result of a regression to the mean if erectile dysfunction is a fluctuating condition. It may also be due to the Hawthorne effect occurring in the interaction with the physicians during enrolment. To more accurately observe these fluctuations, it would be beneficial to analyze the difference in erectile function changes according to the time of diagnosis (early-stage vs. end-stage).

In this study, we identified four distinct latent groups: “consistently maintained normal erectile function group” (9.7%), “rapidly worsened and then rapidly improved group” (9.2%), “gradually improved in the early stage, and then gradually worsened group” (36.8%), and “consistently maintained poor erectile function group” (44.3%), based on GMM analysis of repeated IIEF-5 measures. This can facilitate our understanding of the characteristics of and differences among patients, which is critical when devising patient-centered care strategies and interventions that are tailored to patients’ needs [18]. We also identified factors that predict which erectile function change group male COPD patients are likely belong to.

Older age, lower economic status, and poorer subjective health status were identified as factors that affect the progression of erectile function in men with COPD. Having patients assess these changes themselves allows them to realistically predict how the level of erectile function will change during the course of their disease. These realistic predictions can lead to self-identification of their current health condition, raise awareness of their subjective health status, and motivate them to utilize resources and implement health behaviors necessary to improve their condition. Subjective symptoms and the perception of health are highly important because they can affect the health status and health-related lifestyle [18, 21]. As outpatient visits should take place on a regular basis due to the nature of COPD, it is also recommended that a cubicle be constructed for patients to evaluate their own levels of sexual health. The cubicle program, such as a Kiosk system, needs to be configured such that most patients are able to respond with a simple electronic format with a touch screen, considering their age. Such a Kiosk system needs to be installed in a separate space in an outpatient clinic or inpatient ward and made of opaque glass walls. This can protect the patient’s privacy and allow for self-assessment of erectile function and sexual health, which patients consider sensitive information, in a more comfortable environment. If it worsens, convey this to the medical personnel and receive sex counseling. They should be guided to increase physical activity in everyday life, to participate in leisure activities in the community, and to make use of available resources, because the subjective health level is affected by these factors. Nurse-led recreational programs for inpatients or group activities among patients with sexual problems are also needed.

Based on the results of this study, we attempted confirm that the health status of patients with COPD changes according to their characteristics over time, even if their baseline conditions are similar when they first visit a medical institution. When healthcare professionals first encounter a COPD patient in the clinical setting, they identify and predict the condition based on the patient’s baseline data. The healthcare professionals’ understanding and awareness of longitudinal changes are highly important for the continuity of care and progress. Therefore, we expected that our study findings would be helpful in planning patient-centered care. For this reason, we analyzed and presented the factors influencing the baseline data in this study. Although this study analyzed longitudinal data collected over 4 years, COPD is a lifelong disease, as it progresses gradually. It is important to observe the progression of health status for a longer period of time, reflecting the progressive nature of COPD. These long-term analyses need to be performed consistently using large-scale national-based data rather than by a single medical institution or individual research team.

Comprehensive assessment and multidimensional monitoring could contribute to the early detection of patients at risk of lower erectile function and QoL, which would allow for earlier intervention. In particular, in Asian cultures including Korea, both medical personnel and patients have a passive attitude toward sexual health problems, making it difficult to implement individualized care for sexual function problems. For policy recommendations, in cases of outpatient departments or inpatient wards that are frequently visited by individuals with chronic diseases, such as COPD, the involvement of sex counseling specialists or trained nurse practitioners could facilitate consultation. Moreover, it is necessary to reduce the burden of medical expenses for patients by converting the medical expenses incurred for the sexual function consultation into medical insurance benefit items. In particular, it seems necessary to link the resources available through consultation for low-income families and collaborate with social work teams.

If the sexual function-related factors identified in this study are used appropriately and systematically clinically, healthcare professionals will be able to evaluate the patient’s sexual health as well as perform regular and continuous monitoring of the respiratory symptoms of COPD patients. It will also provide a basis for decision-making and defining patient outcomes in patient-centered management.

This study had several limitations. First, it was a secondary data analysis using cohort data, and only the data of patients who were registered to the cohort could be analyzed; therefore, there is the possibility of selection bias, and the sample size was limited. Because this study was analyzed using the Korean cohort (KOLD) data that recruited patients with obstructive lung disease, it was not possible to make a comparative analysis with non-COPD groups or healthy general populations. In the future, it will be necessary to carry out a comparative analysis with large nation-based data sets such as the Korean National Health and Nutrition Examination Survey or National Health Insurance. In addition, despite having erectile dysfunction, subjects who were taking medications that affect erectile function or participating in a separate program for sexual functioning were excluded from this study based on the exclusion criteria, considering the influence of exogenous variables. Although some GMM analyses target large numbers of samples using large national or multinational cohort data, some previous GMM analyses have included a smaller number of samples when investigating specific health problems [27]. Due to the limited number of samples registered in the cohort and the limited research variables, the present study did not obtain the recommended number of subjects.

The results of this study may not be generalizable, as the conservative culture of Korea hinders discussion of sexual issues. Furthermore, some factors that can affect patterns of changes in erectile function were not considered. The variation of erectile function among COPD patients may be primarily affected by medication compliance, continuity of follow-up observation, and changes in patient condition, etc. It is necessary to analyze such major situational changes and erectile function levels simultaneously. Future studies should consider various situational variables (time-varying variables) that change with time. In this study, it was not possible to identify whether changes in the patients’ condition, such as changes in the physical functional status, changes in medications and dosage, newly diagnosed diseases, psychological status, economic status, health-related behaviors (e.g., smoking cessation, regular exercise, healthy food consumption, etc.) and life events (e.g., changes in family composition such as separation by death, divorce, etc.), could have affected erectile function. A dynamic model-based analysis is required to reflect the time-varying variables. This requires a much larger sample than that in the present study, and any influencing variables should be identified and systematically planned and collected.

## Conclusion

In this study, the erectile function of 185 men with COPD tended to improve in the first year and then worsen gradually over time despite treatment for COPD. The contributing factors for the differences in baseline erectile function among male COPD patients were older age, poorer economic status, higher BODE index and poorer self-rated health status. Using GMM, we identified four distinct latent groups as follows: “consistently maintained normal erectile function” (9.7%), “rapidly worsened and then rapidly improved” (9.2%), “gradually improved in the early stage and then gradually worsened” (36.8%), and “consistently maintained poor erectile function” (44.3%). Progression of erectile function was significantly associated with age, economic status, and self-rated health status. This study is meaningful, in that it overcame the limitations of the previous cross-sectional studies that considered COPD patients as a homogeneous group, and longitudinally investigated changes in the health status of the participants and identified predictors of erectile dysfunction.

These findings are beneficial for understanding the efficacy of a treatment process and introducing appropriate patient-centered interventions. Physicians and clinical nurses can identify groups at risk of erectile dysfunction and provide appropriate preventive interventions that could reduce the risk or enhance protective factors, which could prevent depression and reduced QoL due to sexual health problems. By identifying diverse personal factors that affect the overall health status, rather than focusing solely on respiratory symptoms, a broader understanding of the chronic illness process can be gained, thereby enhancing the quality of patient-centered care. These strategies may improve the sexual health and QoL of male COPD patients and may contribute to cost-effective and efficient patient care management.

## Data Availability

When receiving the consent received from the the participants, the data are to be used only for the research purposes and are not to be disclosed to public without the approval of the KOLD cohort study group. The datasets used and analysed dursing current study are available from the corresponding author upon reasonable request.

## Abbreviations

6MWD: Six-minute walk distance
AIC: Akaike information criterion
ANOVA: Analysis of variance
ATS: American thoracic society
BAI: Beck anxiety inventory
BDI: Beck depression inventory
BIC: Bayesian information criterion
BMI: Body mass index
BODE: Body mass index, the degree of airflow obstruction, functional dyspnea, exercise capacity
CAT: Chronic obstructive pulmonary disease assessment test
CI: 95% Confidence intervals
COPD: Chronic obstructive pulmonary disease
FEV_1_: Forced expiratory volume in 1 s
FVC: Forced vital capacity
GMM: Growth mixture modeling
GOLD: Global initiative for chronic obstructive lung disease
IIEF-5: International index of erectile function-5
KOLD: Korean obstructive lung disease cohort
mMRC: Modified Medical Research Council
OECD: Organization for economic co-operation and development
OR: Odds ratios
QoL: Quality of life
SD: Standard deviation
SE: Standard error
